# Embracing the “Microbiology+” era

**DOI:** 10.1002/mlf2.70074

**Published:** 2026-02-23

**Authors:** Wei Qian, Jizhong Zhou

**Affiliations:** ^1^ Institute of Microbiology Chinese Academy of Sciences Beijing China; ^2^ Institute for Environmental Genomics University of Oklahoma Norman Oklahoma USA; ^3^ School of Biological Sciences University of Oklahoma Norman Oklahoma USA; ^4^ School of Civil Engineering and Environmental Sciences University of Oklahoma Norman Oklahoma USA; ^5^ School of Computer Sciences University of Oklahoma Norman Oklahoma USA; ^6^ Earth and Environmental Sciences, Lawrence Berkeley National Laboratory Berkeley Califonia USA

The field of life sciences is undergoing a profound revolution. Besides reshaping the landscape of all scientific investigation by artificial intelligence (AI), we have entered into an era of “Microbiology+,” since essential roles played by microbes as the pivotal nexus linking human health, sustainable agriculture, ecological restoration, climate change, and even biomanufacturing are becoming increasingly evident.

Microbiological research is driven both by the curiosity to uncover the fundamental principles of microscopic life and by the imperative to address pressing societal challenges. This dual motivation is embodied in the concept of “Pasteur's Quadrant,” introduced by Donald E. Stokes in 1997, which describes research that simultaneously advances fundamental understanding and is inspired by practical use^1^. Microbiology, perhaps more than many other disciplines, naturally occupies this quadrant, which links discovery‐driven inquiry with tangible benefits for health, industry, and environment. From Louis Pasteur's elucidation of fermentation mysteries and foundational principles of vaccination, to Alexander Fleming's serendipitous discovery of penicillin ushering in the antibiotic age, and the revelation of symbiotic mechanisms in plant root nodule bacteria propelling green agriculture, microbiology has spurred significant contributions toward a better life of mankind in history. Today, propelled by revolutionary advances in research tools and the acceleration of multidisciplinary convergence, microbiology is transcending its traditional confines, manifesting a distinctive “Microbiology+” character: microbial influence radiates across medicine, agriculture, environmental sciences, materials, evolution, climate change, and data sciences, which position microbiology as the critical node connecting disparate branches of life sciences and the vital springhead of innovation.

The driving force behind “Microbiology+” lies in the staggering diversity and evolutionary depth of the microbial world, which represents an immense, largely uncharted frontier of life that offers virtually limitless opportunities for discovery and integration across disciplines. The true extent of microbial diversity on Earth remains unknown, with estimates reaching as high as one trillion species^2^. Beyond sheer numbers lies an even greater expanse of metabolic innovation, ecological interactions, and adaptive strategies. Together, this hidden biosphere constitutes one of the planet's greatest reservoirs of biological novelty, holding transformative potential for science, technology, and society. This unparalleled diversity endows microbial research with an intrinsic “permeability”: environmental engineers harness microbes to remediate contaminated soils and waters; agronomists modulate rhizosphere microbiomes to enhance crop resilience and nutrient efficiency; and synthetic biologists modify microbes as “cellular factories” for high‐value compound production. Especially, recent microbiome investigation revealed the gut microbiota's profound impacts on immunity, nervous system, and metabolic health. Microbiome research has propelled microbiology from the scrutiny of singular pathogens or functional strains toward a holistic apprehension of “holobiont” and their interaction networks with various organisms. This paradigmatic migration, from “individuals” to “communities,” from “static taxonomy” to “dynamic functionality,” empowers microbiology to interface more precisely with its applications such as precision medicine, ecological remediation, and sustainable agriculture.

To embrace the “Microbiology+” era, we must revitalize and innovate foundational microbiological research, particularly by overcoming the problem of “unculturable microbes.” Presently, a small portion of environmental microbial species can be laboratory‐cultured. The development of “culturomics,” which leverages simulated niches, microfluidic chips, co‐cultivation strategies, and data prediction technology, will illuminate this “microbial dark matter,” unlocking novel genetic reservoirs, metabolic routes, and ecological roles. Only by integrating a broader microbial cohort into axenic culture can we dissect their physio‐biochemical intricacies, thereby laying an unassailable groundwork for engineered applications. In addition, microbiologists must proactively embrace profound collisions and fusions with AI, physics, chemistry, materials science, and so forth. Microbial research produces oceans of data, with which machine learning can parse multiple omics datasets to forecast microbial functions, interactions, and even bespoke microbial architectures. The intertwining of AI and microbiology will undoubtedly lead the future investigation and development of biotechnology in the coming decade.

The “Microbiology+” era is approaching. mLife, together with other excellent academic journals, will serve as a platform for researchers to inspire their creative ideas. It will publish more thought‐provoking fundamental research findings, more studies that leverage interdisciplinary science to address classic questions, and more novel concepts and theories for debate. Through these efforts, mLife will strive to leave its mark on this exciting era.
